# 

**Published:** 1891-04-04

**Authors:** 


### NOTICE TO CORRESPONDENTS.

All MS., Letters, Books for Review, and other matters intended for the Editor should be addressed The Editor, The Lodge, Porchester Square, London, W.

The Editor cannot undertake to return rejected M.S., even when accompanied by stamped directed envelope.

**Notice to Advertisers and Subscribers.**

All Advertisements, Orders for Copies of The Hospital, and Business Communications should be addressed to The Publisher, *not to the Editor*, The Hospital, 140, Strand, W.C.

The Cost of Subscription, post free, is as follows:—Per week, 1½d.; per quarter, 1s. 8d.; per half-year, 3s. 3d.; per annum, 6s. 6d. Foreign:—Per annum, 8s. 8d.; payable, in all cases, in advance.

### General Charities.

[This Column is devoted to an account of work of charitable institutions other than Medical Charities. The Editor will be glad to receive reports or news of work at 140, Strand. *Philanthropy* should be plainly written on the envelope.]

Of the smaller charitable institutions in this country few do more useful and valuable work than the **AFTER-CARE ASSOCIATION** for poor and friendless female convalescents on leaving asylums for the insane. The Association boasts, and we believe correctly, that it is the only one in this country which offers the special help afforded by it. Many of those who come within the scope of its operations are placed in cottage homes which, it has been found by experience, the women much prefer to large institutions, others are helped by grants of clothing or money, and those who do not come within the rules are assisted to obtain relief in other channels. During the last financial year sixty-six cases were brought before the Committee as against fifty in the previous year, and of these fifty-six were accepted. By means of this Association many unfortunate people are enabled once more to gather together the strength which is so much needed in the struggle for life, who without this help must inevitably sink. Donations in aid of the work will be gratefully acknowledged by the Secretary, Mr. H. Thornhill Roxby, Arden Lea, Walthamstow, Essex.

Owing to the continued distress amongst many of the clergy, more especially those whose income is mainly derived from tithes, which seems to increase, notwithstanding the general improvement in trade, the **CORPORATION OF THE SONS OF THE CLERGY** is compelled to renew the appeal which was made last year, at the suggestion of the Archbishop of Canterbury, for special contributions towards alleviating much unmerited poverty. There are many well-to-do clergymen who can and should show active and practical sympathy with their less fortunately placed brethren. A decrease of twenty-five per cent. in the income of a comparatively rich man is not a pleasant circumstance to face, and to a man of very limited and fixed income such a reduction means not only the giving up of luxuries and comforts, but too often absolute inability to pay his way and consequent penury. One hundred pounds of tithe will this year bring in only £76 3s. 3d., but the fall in tithe is not the only misfortune to be battled against. The cry that the glebe has fallen out of cultivation and is tenantless is only too familiar to those who are intimately acquainted with the pressing needs of a hard-working section of society. Annual subscriptions, donations for a term of years, or contributions in any form and of any amount will be gratefully acknowledged by the Registrar, Mr. W. Paget Bowman, Corporation House, Bloomsbury Place, W.C.

With the month of March the season of anniversary dinners of the numerous charitable institutions in this country begins, with the inevitable appeal to those who are blessed with the good things of this world to show in a practical manner by loosening their purse strings sympathy for those whose strength has proved unequal for the struggle for life. Public dinners are found to be the best means for gaining this desirable end, and there is now scarcely a single charity of importance which does not derive its principal means of support from this source rather than from annual subscriptions. As the claims and needs of the unfortunate are ever with us, so the presence of the inevitable festival dinner seems destined to haunt the dreams of the charitably disposed for at least four months in the year. No doubt in sheer desperation, and recognising to the full the necessity of obeying the law of self-preservation, many people have, with benefit to themselves if not to institutions, made it a rule never to attend public dinners. The dinner of the **NEWS-VENDORS' BENEVOLENT AND PROVIDENT INSTITUTION** was held on the anniversary of the publication of the first daily newspaper in London, which took place on March 11th, 1702. As the Chairman pointed out, the calling of newsvendors is of such a character that they are peculiarly exposed to all the illnesses resulting from exposure to our changeable climate. The result of the Chairman's appeal was the addition of nearly £800 to the funds of the society, a large proportion of which is devoted to the assistance of the widows of newsvendors.

At the annual general meeting of the **ROYAL LITERARY FUND** held at Adelphi Terrace on the 11th ult., the Earl of Derby, the President of the Corporation, congratulated the members on the satisfactory condition of the fund. This favourable position is in no small degree due to the great success of the last two anniversaries, and a continuance of prosperity will much depend on the success of its future festivals. The receipts on the occasion of the centenary last year, when the Prince of Wales presided, amounted to nearly £4,300. Yet notwithstanding the fact that the President is able to congratulate the members that the fund is prosperous, it cannot be said that an assured income of barely £2,000 a-year is a large one for such an institution. Without the receipts from the annual dinner the work of the administrators would practically be crippled. The Lord Chancellor will take the chair at the dinner to be held on May 6th, and the names of gentlemen who are willing to act as stewards will be gladly received by the Secretary, 7, Adelphi Terrace, W.C.

### Scraps and Gleanings.

The Seaside House Convalescent Home, Whitby, will be reopened on April 7th.

A rich native of Bombay has founded an asylum for female lunatics in that city.

Sir Spencer Wells has been elected a Foreign Associate of the French Academy of Medicine.

A bazaar has been held in the Rotunda, Dublin, in aid of the funds of the Dublin Female Hospital.

It has been discovered that skeletons are among the new necessaries of life not taxed by the McKinley Tariff.

The proceeds of the amateur theatricals which were given in the Queen's Theatre, Dublin, will be handed over to the Orthopædic Hospital.

The Worshipful Company of Mercers have made a grant of £500 to the "Buildings Completion Fund" of the Royal Free Hospital, Gray's Inn Road.

The doctors in Berlin are henceforth to wear white hats, so that they may be immediately recognised in the street by anyone desiring medical assistance.

The inquiry into the sanitary condition of St. Bartholomew's Hospital will begin soon after the Easter holidays. The proceedings will be private, but the report will be public.

At a meeting of the Church of England Working Men's Society, the Dean of Rochester reported that they were now free from debt, and it only remained to unite for future success.

Mrs. Reynard, who died recently, has left all her wines to the Willingham Cottage Hospital, which she established and endowed. Her will contains many other charitable bequests.

The influenza is spreading in an alarming degree in Chicago. The death-rate has increased to 150 daily. Ten thousand cases are reported from Pittsburg and 2,000 from Cleveland (Ohio).

Dr. G. H. Humphreys has resigned his position as Resident Medical Superintendent at the Bradford Fever Hospital, where he has worked indefatigably to forward the interests of the institution.

An old lady who is in receipt of 5s. a-week relief money from the City of London Union, believes that she is heir to legacy of £15,000, and she has applied to the Board to assist her with a little money to prosecute her claim.

The Delancey Fever Hospital issues a good report; they have treated 115 cases of scarlet fever during the year, and there was only one death. The trustees have had to withdraw £150 from capital to meet the current expenses of the year.

The Duchess of Teck attended the meeting of the Richmond Aid Committee of the National Society for the Prevention of Cruelty to Children, which was held at Buccleuch House on March 25th. The chair was taken by Sir Whittaker Ellis.

The financial statement in the report for 1890 of the Aberdeen Hospital for Sick Children shows that the income from all sources was £1,613 16s. 0½d., as against £1,717 14s. 3d. in 1889, and the expenditure £1,717 8s., as compared with £1,637 9s. 10½d.

At Chester the Duke of Westminster has nominated a lady as a candidate for election on the Board of Guardians. No lady having ever sat on the Board before, considerable interest has been caused by the Duke's action, and the lady was returned unopposed.

The twenty-second annual report of the Mildenhall Cottage Hospital states that the number of patients treated in 1890 was 43 as against 33 in 1889. The receipts amounted to £324 9s. 8d., and the expenditure to £326 17s. The hospital has received many useful gifts during the year.

Miss Alice McLaren, a Scotch lady, who has studied at the London School of Medicine, has just been appointed House-surgeon of Leith Hospital, and Mrs. Wilson, a student of the Woman's Medical School, has been appointed House-surgeon at the Templars' Hospital, Hampstead.

The fiftieth annual report of the Queen's Hospital, Birmingham, shows the financial position of the hospital to be thoroughly satisfactory. Owing chiefly to the exceptional amount received in legacies during the year, there is a balance of income over expenditure of £2,491 16s. 8d.

On March 18th Mr. John Ellison, Chairman of the West Derby Guardians, laid the foundation stone of the new Infirmary and Nurses' Home, to be erected in Mill Road for the purposes of the West Derby Union. The old building, which the new one is to replace, was erected about fifty years ago.

The twenty-fifth annual report of the Bristol Royal Hospital for Children and Women states that the total expenditure for 1890 was £3,845 18s. 5d., while the income was £3,354 18s., leaving a deficiency on the year of £491 0s. 5d. To meet this deficit the Committee sold out a portion of their investments.

The report for 1890 of the General Hospital, Cheltenham, shows the past year to have been satisfactory, but uneventful. The income was £228 16s. 5d. in excess of that of 1889, while the expenditure shows an increase of £7 3s. 10d. compared with outlay in 1889. The Hospital Trust Funds have been re-invested, and the increased dividend derived from this transfer will amount to £65 1s. 4d. per annum.

A clergyman has been summoned in Croydon to show cause why he should not have his daughter, aged two years, vaccinated. The defendant said that he had conscientious objections to the operation being performed. He pleaded for his defence that the matter is at present "sub judice." The Mayor made an order on the defendant to have his child vaccinated within a month, and to pay 6s. 6d. costs.

At the annual general court of the North London Hospital for Consumption, it was stated that one of the staff had been sent to Berlin to study Dr. Koch's system for the treatment of tuberculosis. Several of the hospital patients had, at their own request, been inoculated with Dr. Koch's lymph, and satisfactory results were reported, but the medical staff thought it too soon to express an opinion as to the permanency of the results.

### BOOKS RECEIVED.

Swan Sonnenschein and Co.

"The New York State Reformatory in Elmira." Alexander Winter, F.S.S. Price 2s. 6d.

"Crime and its Causes." By William Douglas Morrison. Price 2s. 6d.

Wright and Co. (Bristol).

"Our Baby." Mrs. Langton Hewer. Price 1s. 6d.

"Lectures on Diabetes." Robert Saundby, M.D.

### The Student of Medicine.

[All communications for this Supplement should be addressed to The Editor of The Hospital, at 140, Strand, with the words, "Students' Column," written in the left hand top corner of the envelope.]

### EDITORIAL NOTES.

The Inter-Hospital Association Cup tie has been decided. St. Bartholomew's, the holders, have been beaten by St. Thomas's by three goals to none.

The Rugby Challenge Cup Competition, on the other hand, is by no means finished. Another drawn game has been played, this time between Middlesex and Guy's, who tied with a try each. This semi-final round has been again redrawn, and now St. Thomas's have to play Guy's and Middlesex have a bye.

Bart.'s had been the holders for two consecutive years, and previous to this Guy's held the cup four years running. Thomas's now hold it for the first time.

Lady Students.—The lady students are not apparently going to have such an unopposed course of clinical instruction in the wards of the Edinburgh Infirmary as our correspondent led us at one time to suppose. The medical staff of the infirmary have conceived of difficulties. In the first place they say that the number of students is already too great for clinical teaching to be conducted in the most satisfactory manner. The staff maintain that they would have to visit the wards twice daily for clinical purposes to teach many students, and that this would not be to the advantage of the patients. A memorial is now started by the present gentlemen students urging the managers of the infirmary to reconsider their decision of admitting the women students. It will be interesting to see if the managers give way to popular jealousies and sentimentalities.

Presentation.—At the Westminster Hospital Medical School, on Friday, March 20th, Mr. James Black, Lecturer in Anatomy, was presented with a marble clock bearing the following inscription: Presented to James Black, Esq., B.A., M.B., F.R.C.S., &c., as a token of esteem and gratitude from his Anatomy Class, Westminster Hospital, 1889-91. Messrs. Cotton and Nitch-Smith were instructed with the arrangements.

Mr. Frederick Gowland Hopkins, B.Sc.Lond., has been awarded the studentship in pathology at Guy's Hospital, recently established by Sir Cameron Gull, in memory of his father, the late Sir William Gull.

### NOTES FROM THE SCHOOLS.

### University of Cambridge.

At Gonville and Caius College a Shuttleworth scholarship open to medical students of the University of not less than eight terms' standing has been awarded to Francis Carr Bottomley, scholar of the college. The value of the scholarship is about £56 per annum, and it is tenable for three years, being given for proficiency in botany and comparative anatomy.

### University of Oxford.

In a congregation held on the 21st inst. the degree of M.D. was conferred on Frederick J. Smith, Balliol.

### Guy's Medical School.

Guy's held a very successful smoking concert on Saturday, the 7th, in the club smoking room. Mr. A. E. Durham, surgeon to the hospital, was in the chair. The chairman was supported by Dr. Hale White and Mr. Clement Lucas, together with several old Guy's men. The proceedings were opened by Messrs. Coulson and Spurrell with "Love and War." Mr. Coulson's "Cupid" was also a great success. Mr. Elsmore with his rendering of Rubinstein and Raff was much appreciated. Messrs. Spurrell and Coulson obtained well-deserved encores for "Anchor d" and "Where'er you Walk." Mr. Wohlmann scored with his song "In Sheltered Vale," and the chairman's exposition of "The Noble Ritter Hugo and the Wiles of the Eve-like Maiden" was much appreciated. A charming song was that of Messrs. Coulson and Alston, entitled, "Go, Pretty Rose," and the chorus of Mr. Bendell's "To-morrow will be Friday" was lustily sung. The second half of the programme was opened by Mr. Lang with a topical song on the Dental School, which was greatly applauded. Mr. Morton then sang "Simply a matter of Business," and Mr. A. Allport followed with "Sister Mary." The evening closed with "Auld Lang Syne." The accompanists of the songs of the evening were Messrs. Bryant, Mackenson, and Cadell, all of whom played a very important part in the satisfactory carrying out of the programme.

The Hospital Advertiser.

### THE STUDENT OF MEDICINE—(*continued*).

### STUDENTS' MEDICAL SOCIETIES.

#### Middlesex Hospital Medical Society.

On Thursday, February 12th, Dr. W. Essex Wynter read a paper entitled "The Value of Pathological Research in the Diagnosis and Prognosis of Living Cases." In commencing, he regretted that more systematic observations were not made by the clerks in the wards, and then showed how much valuable information could be gained, and with but slight expenditure of time, by the daily examination of sputa in the cases under observation, and also by the more frequent use of the ophthalmoscope, sphygmograph, hæmocytometer, and hæmoglobinometer. Dr. Wynter discussed very carefully the processes in vogue for examining sputum, showing how such an examination was of the greatest assistance both in diagnosis and prognosis, and urged upon every student the advantages to be gained by making sketches of the appearances revealed by the microscope. In conclusion, he pointed out the more important facts obtainable by the daily examination of vomit and urine. Mr. Leopold Hudson argued that pathology was the most valuable of all subjects, and that it was the most neglected; that men read for examinations only, and without understanding thoroughly what they read, with the result that they failed in pathology. Dr. Coupland regretted that the architectural arrangements of the hospital did not, at present, favour such thorough and daily examinations as he would wish to see the students engaged in; but he hoped for better times. Mr. Rodd pointed out the value of electricity in diagnosis of many nervous diseases; and Mr. Helby and Mr. Holding also took part in the discussion. Dr. Wynter then replied tothe various points that had been raised, and brought the meeting to a close with a microscopical demonstration of some of the larger parasites.

#### Westminster Hospital Medical School.

☞ About fifty members were present at the monthly meeting of the Guthrie Society. Mr. C. Stonham presided. Mr. W. G. Spencer read the second part of his paper on "Some Objections to Darwinism." He divided his lecture into three heads as follows: 1. Objections furnished by geology. The primeval rocks contained no traces of life, but in the Cambrian rocks there were specimens of all the invertebrate classes. In the very lowest layers there were animals with perfect insect eyes. Where were all the intermediate types? In the Devonian period fish were found, but no animals at all approaching fish were found in the lower layers. In the coal were found fossils of specimens of mimicry much larger than any found now. In the triassic and jurassic layer lizards were found, there being no forms in lower layers. 2. The evidence afforded by the geographical distribution of animals. In the West Indian Islands there was neither jaguar or monkey as was found in South America. In Madagascar there were lemurs (which occupy a space midway between insectivors and apes). How insectivors are confined to Asia, and monkeys were only found in one or two places in Africa. 3. Ancient man. Human skulls are divided into long-headed, where the relation of breadth to length is less than 80 to 100, and short-headed, where the ratio is greater than 80 to 100. The earliest races, as shown by the skulls found on borders of the Rhine, were long-headed whereas apes are short-headed, merely appearing long-headed from the size of their frontal sinuses. Mr. Spencer's paper, which was about forty minutes' duration, was not followed by much discussion, the subject evidently being somewhat abstruse for the ordinary student. Mr. W. Lewis then read a paper on "Lectures: Their Use and Abuse," which excited the oppressed and overworked medical students present into somewhat loud and denunciatory language regarding the length and number of lectures they were forced to attend.

### APPOINTMENTS.

At Guy's Hospital, G. H. S. Daniell, M.B., B.C. Cantab., and F. G. Swayne, M.B., B.C. Cantab., have been appointed house surgeons; J. Fawcett, M.B., B.S. Lond., and H. W. Webber, M.B., B.S. Lond., house physicians; and J. S. Richards, M.B., B.S. Lond., assistant house physician. At the Dental Hospital of London, E. H. L. Briault, L.D.S. Eng., Arthur Collyer, L.D.S. Eng., W. H. Delamore, L.D.S. Eng., and J. Percy Smith, L.D.S. Eng., have been appointed demonstrators. At the Brown Animal Sanatory Institution, Charles S. Sherrington, M.A., M.B. Cantab., has been appointed professor-superintendent. At the Royal Academy of Arts, William Anderson, F.R.C.S. Eng., L.R.C.P. Lond., has been appointed Professor of Anatomy. At the North-Eastern Hospital for Children, R. A. Dunn, M.R.C.S., L.R.C.P. has been appointed junior house surgeon. At the Leith Hospital, Alice J. Maclaren, M.B. Lond., has been appointed house physician. At the Barnes Convalescent Hospital,

### The Hospital Advertiser.

THE STUDENT OF MEDICINE—(*continued*).

Cheadle, John Edward Platt, M.D. Lond., M.R.C.S., L.R.C.P., has been appointed resident medical officer. At the Miller Hospital, Patrick Cumin Scott, B.A., M.B. Cantab., M.R.C.S., has been appointed physician. At the Aberdeen General Dispensary, A. T. G. Scott, M.A., M.B., C.M. Aberd., has been appointed medical officer. At the Ear and Throat Dispensary, Edinburgh, Alexander Black, M.B., C.M., F.R.C.P. Edin., has been appointed surgeon. At St. Peter's Hospital for Stone and Urinary Diseases, Francis X. Da Costa, F.R.C.S. Eng., L.R.C.P., has been appointed house surgeon. At the Victoria Dock Dispensary, D. N. B. Emerson, B.A., M.B., B.Ch., B.A.O. Dubl., has been appointed senior resident medical officer. At the Kilmarnock Infirmary, William Frew, M.D. Edin., has been appointed physician. At the Bear Yard Workhouse, Strand Union, John Russell Harris, M.D. Brux., has been appointed medical officer. At the Sick Children's Hospital, Edinburgh, Henry Grenville Huie, M.B., C.M., and R. B. Rudolph, M.B., C.M., have been appointed resident physicians. At the Bristol General Hospital, Francis Poole Lansdown, M.R.C.S. Eng., L.S.A., has been appointed surgeon. At the Coton Hill Hospital for the Insane, Stafford, L. H. Liston, M.R.C.S., L.R.C.P., L.S.A., has been appointed assistant medical officer. At the Hertford County and City Lunatic Asylum, C. S. Morrison, L.R.C.P., L.R.C.S. Edin., has been appointed assistant medical officer. At the Royal Infirmary, Manchester, Ernest Septimus Reynolds, M.D. Lond., M.R.C.S. Eng., has been appointed superintendent medical officer. At the Birmingham Eye Hospital, James William Russell, M.A., M.B., B.C. Cantab., has been appointed medical officer. At the Royal Portsmouth and Portsea and Gosport Hospital, Edward James Wallace, M.D., C.M. Glas., has been appointed honorary physician. At the Home and Infirmary for Sick Children and South London Dispensary for Women, Lower Sydenham, F. J. Lorimer Hart, M.B., C.M., L.M.Edin., has been appointed honorary assistant medical officer.

### VACANCIES.

The following vacancies are announced this week, the amount of salary in each case being given after the name of the institution:

**Resident Medical Officers.**

Birmingham City Asylum, Rubery Hill, near Bromsgrove, Clinical Assistant—Board, &c.
Bradford Children's Hospital, House Surgeon—Salary £80 and Board, &c.
Borough Lunatic Asylum, Portsmouth, Clinical Assistant—Board.
Bridgewater Infirmary, House Surgeon—Salary £80.
County Asylum, Shrewsbury, Junior Assistant Medical Officer—Salary £100 and Board, &c.
County Asylum, Rainhill, near Liverpool, Assistant Medical Officer—Salary £100, Board, &c.
Holloway Sanatorium (Hospital for the Insane), Clinical Assistant—Board, &c.
Kent County Asylum, Chartham, near Canterbury, Junior Assistant Medical Officer—Salary £125 and Board, &c.
Paddington Infirmary, Resident Clinical Assistant—Honorarium and Board, &c.
St. Marylebone General Dispensary, Welbeck Street, W., Resident Medical Officer—Salary £105.
Salford Union, Assistant Medical Officer—Salary £130 and Board, &c.
Stockport Infirmary, Assistant House Surgeon—Board, &c.
South Devon and East Cornwall Hospital, Plymouth, Assistant House Surgeon.
Worcester County and City Lunatic Asylum, Third Assistant Medical Officer—Salary £100 and Board, &c.
York Lunatic Asylum, Assistant Resident Medical Officer—Salary £100 and Board, &c.

**Non-Resident Medical Officers.**

Burton-on-Trent Friendly Medical Association, Junior Medical Officer.
Cancer Hospital, Fulham Road, Assistant Surgeon.
Charing Cross Hospital, Surgical Registrar—Salary £40.
Charing Cross Hospital Medical School, Demonstratorship of Physiology Honorarium £100.
London Lock Hospital—Registrar.
Queen's College, Birmingham, Professor of Medicine.
Royal Victoria Patriotic Asylum for Girls, Wandsworth Common, Medical Officer—Salary £100.
St. Mary's Hospital, Surgeon.
Royal College of Physicians, Milroy Lecturer.
West London Hospital, Hammersmith Road, Assistant Surgeon.

### PASS LISTS.

**Victoria University Examination Lists.**

Faculty of Medicine.—Second Examination.—First Division.—H. Ainsworth, E. L. Compston, R. S. Hardman, and P. M'Dougall (Owens College), D. Seaton and W. W. Stoney (Yorkshire College), and P. Thompson (Owens College). Second Division.—J. R. Alcock, H. C. Cadman, D. H. Cheetham, E. A. Goulden, and E. Harrison (Owens College), S. H. House (University College), J. Jones and A. Leigh (Owens

### The Hospital Advertiser.

THE STUDENT OF MEDICINE—(*continued*).

College), E. S. Miller and J. P. Nixon (University College), G. M. Y. Whittingham (Owens College), and R. L. Wood (University College).

Distinguished in Anatomy.—H. Ainsworth and P. Thompson (Owens College).

Final Examination, Part I.—The following have satisfied the examiners: G. F. Chadwick, J. G. Clegg, S. Crawshaw, A. J. Edwards (Owens College), A. Harris and S. R. Knight (University College), R. W. Marsden, G. Stowell, J. H. Taylor, J. H. L. Tylecote, W. H. Waddington, and W. B. Warrington (Owens College).

Final Examination, Part II.—First Division.—H. A. Beaver (University College) and W. Griffith (Owens College). Second Division.—J. H. Bailey (Owens College), S. H. Fairrie (University College), J. J. Howorth, C. K. Rawes, and F. Robinson (Owens College).

### ATHLETICS.—Football.

Inter-Hospital Association Challenge Cup (Final tie).—St. Bartholomew's (holders) v. St. Thomas's.—The final tie for the Association Cup was played off on Wednesday, the 25th, after two postponements and one drawn game, with the result that the holders were beaten by Thomas's by three goals to none. The ball was set rolling at a quarter past three by Harward for Thomas's, who started with the wind in their faces. Barraclough and Slocock at once went back. The latter being cleverly deprived by Fernie, Henry with a long pass sent on to Hogarth, who, after a good run, sent the ball over the cross bar. From the goal kick Thomas's attacked all along the line, but failed to break through their opponents' second line, and Lewarne with a long kick cleared. Fox then got possession of the ball, and with Dixon managed to dribble the ball down. Saunders, however, proved impassable, and a further attack by the Surrey side resulted in Barraclough scoring off a pass from Slocock within fifteen minutes from the commencement of the game. The ball being again sent off from the centre, Bart.'s right wing gave way, and a corner was forced off Lever. Mason cleared, but before the ball could be returned Dixon sent in a splendid shot for goal. Grant-Wilson saved, at the expense of another corner, which was again unproductive. Another opportunity presenting itself, Wilson sent in a slow shot, but this was put off by the Thomas's custodian. At this point of the game Barraclough was very prominent, but being ill-supported, was stopped by Barker, who crossed to the right. Then Fernie, after a brilliant single-handed run, very nearly proved himself too much for Grant-Wilson with a tall shot on the side. Next the opposite side attacked. Sound tackling by Lewarne and Barker alone prevented them getting through. At length the pressure was relieved by Hogarth, who had the goal open when he recovered the ball. Immediately following this Dixon forced an unproductive corner off Grant-Wilson. From this point up to half time the play was very fast, and the holders had the best of it, but failed to equalise. At half time the score was Thomas's one goal, Bart.'s none. At the commencement of the second half of the match Bart.'s went away on the left and centre, Hogarth being neatly robbed by Blount. At this point Thomas's went away, the most conspicuous player being Slocock, but he did not pass well, and Henry returned. Then Barraclough with a long kick sent the ball behind. Shortly afterwards, Barraclough was pulled up by Lewarne. Hogarth passed Saunders, and made for the opponents' goal. Mason ran up just in time to spoil his shot, and the ball went wide of the uprights. The Bart.'s men made several desperate attacks, but always failed to score, the ball going either over or wide of the bar. Towards the end of the game Henry, when clearing, slipped up and handled the ball. Mason took the free kick, and after a warm attack round the goal Barraclough sent the ball past Coulby, and so Thomas's led by two goals. About a minute from the call of "Time!" Saunders headed through from a corner, and Thomas's won a well-fought match by three goals to none. Referee, Mr. E. B. Snooke (Cantab); umpires, Messrs. H. Humphrey and A. C. Friend. Sides:—

St. Thomas's.—C. W. Grant-Wilson (goal), F. W. Mason (captain) and J. W. Laver (backs), G. B. Blount, F. E. Saunders, J. Gilham (half-backs), R. Slocock and H. C. Barraclough (right), H. Harward (centre), J. J. Powell and W. Allen (left).

St. Bartholomew's.—G. A. Coulby (goal), F. Fernie and J. Cropper (backs), F. Lewarne, C. Barker, and E. Henry (half-backs), J. Fernie and N. Wilson (right), F. Dixon (centre), H. Fox and R. G. Hogarth (left).

Previous Winners.

1885. Guy's beat St. Bart.'s by 2 to 1.
1886. Guy's beat St. Bart.'s by 1 to 0.
1887. Guy's beat St. Thomas's by 1 to 0.
1888. Guy's beat St. Bart.'s by 1 to 0.
1889. St. Bart.'s beat Guy's by 4 to 0.
1890. St. Bart.'s beat St. Thomas's by 4 to 1.
1891. St. Thomas's beat St. Bart.'s by 3 to 0.

### Racquets.

*Oxford* v. *Cambridge*.—The Queen's Club was well filled with visitors on Tuesday, the 31st, to witness the double-handed match between the two Universities, the following being the players:—E. L. Metcalfe, Eton and Christchurch, and F. S. Cockayne, Charterhouse and Oriel, representing Oxford; P. Ashworth, Harrow and Trinity, and E. H. Miles, Marlborough and King's, representing Cambridge. Ashworth won the spin of the racquet and opened the service. Cambridge won the first game at 15-3 in eight minutes. In the second the Cantabs ended their fourth hand at 3-2 and Oxford ran out. At the end of the fourth innings, Metcalfe having won the game for Oxford, the score stood "2 all." Afterwards with the score at "3 all" Ashworth opened the deciding game, but he was ousted after he had scored a single. Oxford eventually won by four games to three. Mr. A. J. Webb was referee, and Major Spens and Mr. E. H. Buckland were umpires. Cambridge have now won 21 of the 35 matches and Oxford 14. This victory for the dark blues is the first they have gained since 1875.

